# 16.1 EPIGENETIC SIGNATURES OF CHILDHOOD AND ADOLESCENT VICTIMISATION USING A GENETICALLY SENSITIVE LONGITUDINAL COHORT STUDY.

**DOI:** 10.1093/schbul/sby014.060

**Published:** 2018-04-01

**Authors:** Helen Fisher, Sarah Marzi, Louise Arseneault, Chloe Wong, Radhika Kandaswamy, Terrie Moffitt, Susanna Roberts, Jonathan Mill, Avshalom Caspi

**Affiliations:** 1 Institute of Psychiatry, Psychology & Neuroscience, King’s College London; 2 King’s College London; 3 King’s College London, Duke University; 4 Exeter University

## Abstract

**Background:**

Stress is a normal, adaptive response to stressors (e.g. events that make a person feel threatened or upset). However, healthy development can be derailed by excessive or prolonged exposure to stress especially during important developmental periods such as childhood and adolescence. Exposure to severe stress may have immediate as well as long-lasting damaging effects on learning, behaviour, and health, and has been implicated in the development of psychosis. One way these may occur is via changes to epigenetic processes (e.g. alterations in DNA methylation). Initial studies have shown that individuals exposed to severe psychosocial stress have different patterns of DNA methylation (epigenetic ‘signatures’) compared to individuals exposed to no/minimal stressful life events, but these studies are methodologically limited.

**Methods:**

This paper describes our examination of the potential link between exposure to multiple forms of severe victimisation (poly-victimisation) during childhood adolescence and DNA methylation differences utilising data from the Environmental Risk (E-Risk) Longitudinal Twin Study, an epidemiological study of 2,232 children (1,116 twin pairs) born in 1994–1995 in England and Wales and followed to 18 years of age (with 93% retention). Multiple forms of victimisation were ascertained in childhood and adolescence (including physical, sexual and emotional abuse, neglect, exposure to intimate-partner violence, bullying, cyber- and crime victimization) by combining the best practices in survey research with comprehensive interview-based approaches. Whole blood samples were collected from participants at age 18, and the extracted DNA was used to quantify genome-wide patterns of DNA methylation.

**Results:**

Epigenome-wide analyses of poly-victimisation across childhood and adolescence revealed several significant associations with DNA methylation in peripheral blood at age 18 years, but these were confounded by tobacco smoking and/or did not survive co-twin control tests. Secondary analyses of specific forms of victimisation revealed sparse associations with DNA methylation that did not replicate across different operationalisations of the same putative victimization experience. Hypothesis-driven analyses of six candidate genes in the stress response (NR3C1, FKBP5, BDNF, AVP, CRHR1, SLC6A4) did not reveal predicted associations with DNA methylation in probes annotated to these genes.

**Discussion:**

Findings from this epidemiological analysis of the epigenetic effects of early-life stress do not support the hypothesis of robust changes in DNA methylation in victimised young people. It is possible that epigenetic epidemiology is not yet well matched to experimental, non-human models in uncovering the biological embedding of stress.

